# Molecular profiles of response to neoadjuvant chemoradiotherapy in oesophageal cancers to develop personalized treatment strategies

**DOI:** 10.1002/1878-0261.12907

**Published:** 2021-02-23

**Authors:** Leonie K. de Klerk, Ruben S. A. Goedegebuure, Nicole C. T. van Grieken, Johanna W. van Sandick, Annemieke Cats, Jurrien Stiekema, Rosa T. van der Kaaij, Arantza Farina Sarasqueta, Manon van Engeland, Maarten A. J. M. Jacobs, Roy L. J. van Wanrooij, Donald L. van der Peet, Aaron R. Thorner, Henk M. W. Verheul, Victor L. J. L. Thijssen, Adam J. Bass, Sarah Derks

**Affiliations:** ^1^ Department of Medical Oncology Cancer Center Amsterdam Amsterdam UMC, location VUmc The Netherlands; ^2^ Department of Medical Oncology Dana‐Farber Cancer Institute Boston MA USA; ^3^ Oncode Institute Utrecht The Netherlands; ^4^ Department of Pathology Cancer Center Amsterdam Amsterdam UMC, location VUmc The Netherlands; ^5^ Department of Surgery Netherlands Cancer Institute/Antoni van Leeuwenhoek Hospital Amsterdam The Netherlands; ^6^ Department of Gastrointestinal Oncology Netherlands Cancer Institute/Antoni van Leeuwenhoek Hospital Amsterdam The Netherlands; ^7^ Department of Pathology Leiden University Medical Center The Netherlands; ^8^ Department of Pathology GROW‐School for Oncology and Developmental Biology Maastricht University Medical Center The Netherlands; ^9^ Department of Gastroenterology and Hepatology Amsterdam UMC, location VUmc The Netherlands; ^10^ Department of Surgery Amsterdam UMC, location VUmc The Netherlands; ^11^ Center for Cancer Genome Discovery Dana‐Farber Cancer Institute Boston MA USA; ^12^ Department of Radiation Oncology Amsterdam UMC, location VUmc The Netherlands; ^13^ Cancer Program The Broad Institute of MIT and Harvard Cambridge MA USA

**Keywords:** chemoradiation, DNA sequencing, gene methylation, genetic biomarkers, oesophageal cancer, predictive markers

## Abstract

Identification of molecular predictive markers of response to neoadjuvant chemoradiation could aid clinical decision‐making in patients with localized oesophageal cancer. Therefore, we subjected pretreatment biopsies of 75 adenocarcinoma (OAC) and 16 squamous cell carcinoma (OSCC) patients to targeted next‐generation DNA sequencing, as well as biopsies of 85 OAC and 20 OSCC patients to promoter methylation analysis of eight GI‐specific genes, and subsequently searched for associations with histopathological response and disease‐free (DFS) and overall survival (OS). Thereby, we found that in OAC, *CSMD1* deletion (8%) and *ETV4* amplification (5%) were associated with a favourable histopathological response, whereas *SMURF1* amplification (5%) and *SMARCA4* mutation (7%) were associated with an unfavourable histopathological response. *KRAS* (15%) and *GATA4* (7%) amplification were associated with shorter OS. In OSCC, *TP63* amplification (25%) and *TFPI2* (10%) gene promoter methylation were associated with an unfavourable histopathological response and shorter DFS (*TP63*) and OS (*TFPI2*), whereas *CDKN2A* deletion (38%) was associated with prolonged OS. In conclusion, this study identified candidate genetic biomarkers associated with response to neoadjuvant chemoradiotherapy in patients with localized oesophageal cancer.

## Graphical abstract

Using DNA sequencing and methylation panels on pretreatment biopsies, we identified candidate molecular alterations associated with response [tumour regression grade (TRG) or survival] to neoadjuvant chemoradiotherapy in localized oesophageal adenocarcinoma (OAC) and squamous cell carcinoma (OSCC). These findings may assist approaches to further individualize oesophageal cancer treatment.

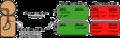

**Abbreviations**
CpG island: stretches of DNA with a high CG:GC ratio, often found and methylated in gene promoters
CRT: chemoradiotherapy
MSP: methylation‐specific polymerase chain reaction
OAC: oesophageal adenocarcinoma
OC: oesophageal carcinoma
OSCC: oesophageal squamous cell carcinoma
PRSC: prognostic score
TRG: tumour regression grade

## 1 Introduction

Oesophageal cancer (OC) is the eighth most common cancer and one of the leading causes of cancer‐related death [1]. Five‐year survival rates are low, mainly because of late‐stage diagnosis and limited effectiveness of systemic therapy [2]. In parallel to developing better therapy for those with more advanced disease, it is important to maximize treatment success in early‐stage disease and thereby prevent disease recurrence. When OC is confined to the oesophagus and regional lymph nodes, treatment is with curative intent. In case of stage II (T1N1M0 or T2N0M0) and III (T2N1M0 or T3‐4aN0‐1M0) disease, neoadjuvant chemoradiotherapy (CRT) with paclitaxel (50 mg·m^−2^), carboplatin (AUC 2 mg·mL^−1^·min^−1^) and concurrent radiotherapy (41.4 Gy in 23 fractions) followed by a surgical resection is a commonly used treatment regimen that improves median overall survival of patients to 49.4 months compared to 24.0 months with surgery alone [3].

OC is classified into two different histological subtypes, squamous cell carcinoma (OSCC) and adenocarcinoma (OAC). OSCC has been shown to be more sensitive to neoadjuvant CRT than OAC; around 49% of OSCC patients have a complete histopathological response (Mandard tumour regression grade (TRG) [4] of 1) compared to only 23% of OAC patients [5]. A complete histopathological response to neoadjuvant treatment is a strong predictor of long‐term survival. Conversely, patients with a limited or absent histopathological response have a comparable survival to patients that underwent a surgical tumour resection without neoadjuvant therapy [6]. As these patients may not benefit from standard neoadjuvant treatment, they may be better treated with alternative neoadjuvant approaches or, alternatively, considered for immediate surgical intervention. At the same time, if it would be possible to predict a complete histopathological response, consideration can be made to forgo surgery, especially in patients with substantial comorbidities or with tumours in locations where the morbidity of resection is higher.

There have been multiple attempts to identify clinical, histopathological and molecular biomarkers for response to neoadjuvant treatment in OC [7], but most studies have been performed in small cohorts and in a focused manner. Irrespective of treatment, recent studies performed by The Cancer Genome Atlas [8] and the International Cancer Genome Consortium [9] identified large genomic heterogeneity within OCs and underlined that OSCC and OAC have profoundly distinct molecular characteristics, both in patterns of somatic mutations and in copy‐number aberrations. OSCC and OAC also differ significantly in DNA methylation patterns [8]. While OSCCs have relatively infrequent DNA CpG island promoter methylation, OACs can be divided into distinct subtypes with a variable degree of CpG island promoter methylation [10]. Whether these molecular characteristics affect response to CRT in OC is currently unknown.

This study aimed to evaluate whether common molecular characteristics are associated with response to neoadjuvant chemoradiotherapy and subsequent survival in OC patients. Thereby, we explore the potential of molecular profiling to complement other clinical and histopathological factors to inform treatment strategies for localized oesophageal cancer.

## 2 Materials and methods

### 2.1 Patient population

Clinical data and pretreatment tissue from 131 patients with stage II–III oesophageal cancer were retrospectively collected from three hospitals (VU University Medical Center, Netherlands Cancer Institute/Antoni van Leeuwenhoek Hospital and Leiden University Medical Center). The study methodology was approved by the ethical committees of all three hospitals and in accordance with the Declaration of Helsinki. Selected patients had been treated with neoadjuvant chemoradiotherapy consisting of paclitaxel (50 mg·m^−2^), carboplatin (AUC 2 mg·mL^−1^·min^−1^) and concurrent radiotherapy (41.4 Gy in 23 fractions) followed by surgical resection. Data on histopathological response, as well as on clinical follow‐up, were documented.

Histopathological response was assessed by pathologists at Amsterdam UMC (NCTG and AFS). Both the ypTNM stage (7th edition), and the Tumour Regression Grade (TRG) according to Mandard [4] were scored. Mandard's TRG consists of 5 tiers, which are TRG1 (no residual cancer), TRG2 (rare residual cancer cells), TRG3 (fibrosis outgrowing residual cancer), TRG4 (residual cancer outgrowing fibrosis) and TRG5 (absence of regressive changes). In addition, we calculated the histopathological prognostic score (PRSC) [11], which is based on ypT stage (ypT0–2 = 1 pt, ypT3–4 = 2 pts), ypN stage (ypN0 = 1 pt, ypN1–3 = 2 pts) and residual tumour per tumour bed (≤ 50% = 1 pt, > 50% = 2 pts) and then divided into three groups (group A: 3 pts total, B: 4–5 pts, C: 6 pts). For the 50% cut‐off for residual tumour per tumour bed, a Mandard TRG up to 3 (‘fibrosis outgrowing residual cancer’) was considered lower than 50%, and a Mandard TRG of 4 (‘residual cancer outgrowing fibrosis’) or higher was considered higher than 50%.

Clinical response was expressed as overall survival (OS) and disease‐free survival (DFS). Survival was defined as time from the date of surgery to death from any cause for OS, and to disease recurrence for DFS. Recurrence was evaluated during standard follow‐up post‐treatment at the surgery department. Recurrent disease was defined as locoregional recurrence or distant metastasis ascertained by radiological or histopathological evaluation. Patients lost to follow‐up were censored at the time of their last contact with the outpatient clinic. Median follow‐up time was 3.7 years (3.7 years for OAC, 4.7 years for OSCC).

### 2.2 DNA extraction

Formalin‐fixed paraffin‐embedded (FFPE) tissue slides were obtained from all patients. An expert pathologist (NCTG) reviewed H&E‐stained sections in order to confirm the diagnosis and to ensure > 50% tumour content in areas for genomic DNA extraction; if necessary, macro‐dissection was performed. From 30 tumours, DNA from adjacent normal oesophageal epithelium was also extracted.

Genomic DNA was extracted from tissue sections using the DNeasy FFPE Tissue Kit (Qiagen, Germantown, MD, USA) according to the manufacturer's instructions with a modification of an overnight incubation with proteinase K. Genomic DNA was eluted into 40 µL total volume and quantified with Quant‐iT PicoGreen DNA assay kit (Invitrogen, Carlsbad, CA, USA) following the manufacturers' instructions.

### 2.3 Targeted sequencing

A total of 200 ng of DNA per sample was fragmented (Covaris sonication, Covaris, Woburn, MA, USA) to 250 bp and purified using Agentcourt AMPure XP beads (Beckman Coulter, Brea, CA, USA). Size distribution after fragmentation was checked using the Agilent 2200 TapeStation system (Agilent Technologies, Santa Clara, CA, USA). To determine the amount of each library to add for sequencing, all libraries were then pooled and low‐depth sequencing was performed on an Illumina MiSeq Nano flow cell (Illumina, San Diego, CA, USA). Concentrations were normalized for analysis based on the number of reads of each adapter barcode. Normalized libraries were again pooled in batches ranging from 12 to 15 samples and enriched for the exonic regions of 243 GI‐specific targets (as previously described [12]) using the Agilent SureSelect Hybrid Capture kit (Agilent). Samples were combined and pooled to a lane equivalent of 32 samples per lane (HiSeq 2500 Rapid Run Mode) for each sequencing pool.

Mutation analysis for single nucleotide variants (SNV) was performed using MuTect v1.1.4 in paired mode using CEPH as a project normal, or the matched normal where appropriate, and annotated by Oncotator [13, 14]. We used the SomaticIndelDetector tool that is part of the GATK for indel calling. Only commonly reported (COSMIC ≥ 3 times), and clear loss‐of‐function mutations were used for analysis.

Copy‐number variants were called using the tool ReCapSeg v1.4.4, which is in development by the Cancer Group at the Broad Institute (https://gatk.broadinstitute.org/). Within the (+) calls, a gene was considered amplified if it had a log_2_ ratio of greater than 2. For loss calls, a gene was considered to have a two‐copy deletion if the log_2_ ratio was less than −0.7.

### 2.4 Methylation‐specific polymerase chain reaction

The methylation status of the CpG island in the promoter region of a GI cancer relevant panel (*CHFR, RASSF1, NDRG4, CDKN2A, MLH1, TFPI2, MGMT* and *RUNX3*) was determined by a two‐step nested methylation‐specific polymerase chain reaction (MSP), as described in detail previously [15]. DNA from normal peripheral lymphocytes from healthy individuals and *in vitro* methylated DNA were included as negative and positive controls.

The methylation index was calculated by dividing the number of methylated gene promoters (ranging from 0 to 8) by the number of successfully tested gene promoters (usually 8).

### 2.5 Statistical analysis

Associations between (epi)genetic events and dichotomized Mandard TRG (TRG1–3 vs 4–5), ypN stage (ypN0 vs ypN1–3) and clinical N stage (0 vs 1–3) and associations between histology and baseline characteristics such as gender and completeness of resection were tested with Fisher's exact test, or, if assumptions were met, a Pearson chi‐squared test (indicated in tables). Associations between (epi)genetic events and TRG, PRSC and clinical N stage, clinical T stage, and between histology and clinical T stage, clinical N stage, ypT stage, ypN stage, TRG and PRSC, and between Mandard TRG and PRSC, were analysed with a linear‐by‐linear exact test. To test associations between methylation index and dichotomized Mandard TRG (TRG1–3 vs 4–5), ypN stage (ypN0 vs ypN1–3), clinical N stage (0 vs 1–3) and histology, a Wilcoxon rank‐sum test was used, and between methylation index and TRG, PRSC, clinical N stage and ypN stage, a Kruskal–Wallis test. Survival differences between binary predictor variables were analysed with a log‐rank test, and Hazard ratio's (HR) calculated with univariate Cox regression analysis. Median follow‐up time was calculated using the reverse Kaplan–Meier approach [16]. The forced entry method was used for both the logistic and Cox multiple regression analyses. *P*‐values (two sided) < 0.05 were considered statistically significant. Multiple comparison correction was performed using the two‐stage linear step‐up procedure of Benjamini, Krieger and Yekutieli, with an FDR (*Q*) of 5%, using graphpad prism (version 8, GraphPad Software, San Diego, CA, USA). All other statistical analyses were performed with spss version 25 (IBM, Armonk, NY, USA). Kaplan–Meier survival plots were generated with the survminer package in r (version 1.1.453) [17].

## 3 Results

### 3.1 Patient characteristics and response evaluation

In our search for molecular biomarkers to tailor treatment decisions in nonmetastatic oesophageal cancer (OC), we isolated DNA from a retrospectively collected series of 131 archival pretreatment tumour biopsies from three different hospitals in the Netherlands. All patients had been clinically diagnosed with stage II or III OC and received treatment with neoadjuvant chemoradiotherapy (CRT), containing carboplatin and paclitaxel, followed by surgical resection. DNA, meeting requirements for targeted sequencing, could be extracted from formalin‐fixed paraffin‐embedded biopsies of 92 out of 131 patients, which included 16 oesophageal squamous cell carcinomas (OSCC), 75 oesophageal adenocarcinomas (OAC) and one undifferentiated carcinoma, and was evaluated using a custom GI‐specific hybrid capture 243 gene panel to assess mutations and copy‐number status, as described before (Table S1) [12]. Baseline patient characteristics are presented in Table 1 and Table S2. The median age at diagnosis was 64 years and patients were predominantly male (78.0%). The majority of patients presented with a ≥ cT3 tumour (82.4%) and/or lymph node positivity (62.6%). Resection of the tumour was complete in 91.2% of cases. OSCC and OAC patients did not differ in pretreatment characteristics (age, gender, T stage, N stage) and completeness of resection (Table 1). Median disease‐free survival (DFS) was 3.2 years and median overall survival (OS) 4.3 years and did not differ significantly between OAC and OSCC (Fig. S1).

**Table 1:** Baseline characteristics of patients whose biopsies were used for the custom upper gastrointestinal cancer‐specific next‐generation targeted sequencing.

	Total	OAC	OSCC	*P*
	*N* = 91 (%)	*N* = 75 (%)	*N* = 16 (%)	
Age at diagnosis				
Median with range	64.0 (37–81)	64.0 (37–81)	65.5 (43–76)	ns
Gender				
Male	71 (78.0%)	61 (81.3%)	10 (62.5%)	ns
Female	20 (22.0%)	14 (18.7%)	6 (37.5%)	
Clinical T stage				
T1	0 (0.0%)	0 (0.0%)	0 (0.0%)	ns
T2	9 (9.9%)	7 (9.3%)	2 (12.5%)	
T3	67 (73.6%)	57 (76.0%)	10 (62.5%)	
T4	8 (8.8%)	5 (6.7%)	3 (18.8%)	
Missing	7 (7.7%)	6 (8.0%)	1 (6.3%)	
Clinical N stage				
N0	28 (30.8%)	25 (33.3%)	3 (18.8%)	ns
N1	37 (40.7%)	32 (42.7%)	5 (31.3%)	
N2	18 (19.8%)	12 (16.0%)	6 (37.5%)	
N3	2 (2.2%)	2 (2.7%)	0 (0.0%)	
Missing	6 (6.6%)	4 (5.3%)	2 (12.5%)	
Completeness of resection				
Complete	83 (91.2%)	69 (92.0%)	14 (87.5%)	ns
Not complete	4 (4.4%)	3 (4.0%)	1 (6.3%)	
Missing	4 (4.4%)	3 (4.0%)	1 (6.3%)	
ypT stage				
ypT0	25 (27.5%)	15 (20.0%)	10 (62.5%)	0.021^a^
ypT1	10 (11.0%)	10 (13.3%)	0 (0%)	
ypT2	7 (7.7%)	7 (9.3%)	0 (0%)	
ypT3	46 (50.5%)	40 (53.3%)	6 (37.5%)	
Missing	3 (3.3%)	3 (4.0%)	0 (0%)	
ypN stage				
ypN0	54 (59.3%)	43 (57.3%)	11 (68.8%)	ns
ypN1	20 (22.0%)	17 (22.7%)	3 (18.8%)	
ypN2	11 (12.1%)	9 (12.0%)	2 (12.5%)	
ypN3	4 (4.4%)	4 (5.3%)	0 (0%)	
Missing	2 (2.2%)	2 (2.7%)	0 (0%)	
Mandard's TRG				
TRG 1	25 (27.5%)	15 (20.0%)	10 (62.5%)	0.004^a^
TRG 2	13 (14.3%)	11 (14.7%)	2 (12.5%)	
TRG 3	22 (24.2%)	20 (26.7%)	2 (12.5%)	
TRG 4	27 (29.7%)	26 (34.7%)	1 (6.3%)	
TRG 5	1 (1.1%)	0 (0.0%)	1 (6.3%)	
Missing	3 (3.3%)	3 (4.0%)	0 (0.0%)	
Prognostic Score				
PRSC A	30 (33.0%)	22 (29.3%)	8 (50.0%)	ns
PRSC B	41 (45.1%)	34 (45.3%)	7 (43.8%)	
PRSC C	17 (18.7%)	16 (21.3%)	1 (6.3%)	
Missing	3 (3.3%)	3 (4.0%)	0 (0.0%)	
Recurrence < 1 year	24 (26.4%)	20 (26.7%)	4 (25.0%)	ns
Median overall survival, years (95% CI)	4.29 (2.9–5.7)	4.29 (3.1–5.5)	3.08 (0.0–6.3)	ns
Median disease‐free survival, years (95% CI)	3.21 (2.3–4.1)	3.53 (2.1–5.0)	2.95 (1.7–4.2)	ns

^a^ Linear‐by‐linear, exact text.

Response to neoadjuvant CRT was evaluated by histopathological tumour regression grading (TRG) using the post‐treatment resection specimen. Tumour regression was graded using the Mandard score, which contains five tiers ranging from 1 (no residual cancer) to 5 (absence of regressive changes) [4]. As expected [3], a complete histopathological response (TRG 1) was observed more often in OSCC patients (62.5%, 10/16) than in OAC patients (20.0%, 15/75; *P* = 0.002; Table 1). The association between higher Mandard TRG scores and shorter disease‐free and overall survival was confirmed (Fig. S2A).

As the Mandard TRG is limited to the response of the primary tumour and does not include response in lymph nodes, we added the Prognostic Score (PRSC) [11] to our outcome measures. The PRSC is a histopathological response grading system that combines tumour regression (≤ 50% vs > 50%) with the presence of residual cancer in lymph nodes (ypN0 vs ypN1–3) and tumour stage (ypT0–2 vs ypT3–4); it ranges from A (favourable prognosis) to C (poor prognosis) [11]. We confirmed a strong association between the PRSC and survival in our series [disease‐free survival (DFS): *P* = 0.0015, overall survival (OS): *P* = 0.0065; Fig. S2B]. Post‐CRT lymph node positivity (ypN) by itself was also a strong predictor of shortened survival as compared to ypN negativity [11] (Fig. S2C). Missing cases excluded, within OAC 30.6% had a PRSC A, 47.2% PRSC B and 22.2% PRSC C; and within OSCC, 50.0% had a PRSC A, 43.8% PRSC B and 6.3% PRSC C.

### 3.2 Genetic alterations in OAC and OSCC

**Fig. 1:**
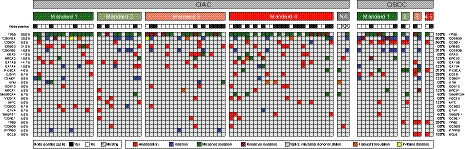
Overview of genomic alterations in relation to histopathological response according to Mandard's tumour regression grade to neoadjuvant chemoradiotherapy in patients with oesophageal adenocarcinoma (OAC) and oesophageal squamous cell carcinoma (OSCC). Percentages indicate frequency of occurrence within OAC and OSCC, respectively.

Targeted sequencing of pretreatment biopsies confirmed known genetic patterns in OAC and OSCC [8]. As expected, *TP53* was the most frequently mutated gene in both OAC (80%, 60/75) and OSCC (75%, 12/16). Other frequently mutated genes were *CDKN2A* (13.3%, 10/75) and *BRCA2* (10.7%, 8/75) in OAC and *PIK3CA* in OSCC (25%, 4/16; Fig. 1, Tables S3 and S4).

Copy‐number variation (CNV) analysis identified amplifications of *ERBB2* (17q12; 20.0%, 15/75), *KRAS* (12p12.1; 14.7%, 11/75) and *GATA6* (18q11.2; 14.7%, 11/75) and deletion of *CDKN2A* (9p21.3; 16.0%, 12/75; Fig. 1 and Table S3) mostly in OAC, while *CCND1* amplification was the most prevalent CNV in OSCC (11q13.3; 56.3%, 9/16). Other commonly observed CNVs in OSCC were deletion of *CDKN2A* and/or *CDKN2B* (9p21.3), amplification of *EGFR* (7p11.2) and amplification of *TP63* [3q28; 25%, 4/16 (all cases with *SOX2* amplification co‐occurred with *TP63* amplification) for (in 2/4 cases with *TP63* amplification, *SOX2* was also amplified); Fig. 1 and Table S4]. These alterations are consistent with the histology‐specific genomic patterns described by The Cancer Genome Atlas [8] and the International Cancer Genome Consortium [9], thereby confirming the feasibility of using a custom‐targeted sequencing panel on archival pretreatment biopsies.

There were no significant associations between any genetic events and clinical N or T stage in both OAC and OSCC. *ATM* mutation was associated with younger age at diagnosis in OAC (median 47 vs 64 years, *P* = 0.031), and *PIK3CA* mutation was associated with younger age at diagnosis in OSCC (median 56 vs 66.5 years, *P* = 0.042).

### 3.3 CpG island promoter methylation in OC

Because CpG island promoter methylation is a common feature of OC [8], we performed a multiplex methylation‐specific PCR on a panel of 8 gene promoters known to be methylated in GI cancers, *CDKN2A*, *CHFR*, *MGMT*, *MLH1*, *NDRG4*, *RASSF1*, *RUNX3* and *TFPI2*, on 105 formalin‐fixed paraffin‐embedded tumour samples, among which 85 OACs and 20 OSCCs. The majority of this group (76/105 samples) had sufficient DNA for both custom GI‐specific targeted sequencing and methylation analyses (Fig. S3).

We confirmed that CpG Island promoter methylation is predominantly a characteristic of OAC, with a median methylation index (promoters methylated/promoters tested) of 0.57 (95% CI 0.52–0.62) compared to 0.25 (95% CI 0.16–0.38) in OSCC (*P* < 0.0001; Fig. S4). CpG island promoter methylation was significantly lower in normal tumour‐adjacent epithelium [mean methylation index 0.05 in normal (*n* = 30) vs 0.51 in tumour; *P* < 0.0001; Table 2].

**Table 2:** Prevalence of promoter CpG island methylation of selected genes in patients with oesophageal cancer.

Gene	Full name	Methylated in normal		Methylated in OAC		Methylated in OSCC		OAC vs OSCC
		*N*/total	%	*N*/total	%	*N*/total	%	*P*
*CDKN2A*	Cyclin‐dependent kinase inhibitor 2A	0/26	0.00	21/66	31.8	5/12	41.7	ns
*CHFR*	Checkpoint with forkhead and ring finger domains	2/30	6.67	46/83	55.4	5/20	25.0	0.015
*MGMT*	O‐6‐methylguanine‐DNA methyltransferase	2/30	6.67	62/85	72.9	12/20	60.0	ns
*MLH1*	mutL homolog 1	0/30	0.00	18/85	21.2	6/20	30.0	ns
*NDRG4*	NDRG family member 4	1/30	3.33	73/85	85.9	1/20	5.0	< 0.001
*RASSF1*	Ras association domain family member 1	1/30	3.33	7/58	12.1	3/20	15.0	ns
*RUNX3*	RUNX family transcription factor 3	4/30	13.33	64/85	75.3	8/20	40.0	0.002
*TFPI2*	Tissue factor pathway inhibitor 2	1/28	3.57	68/85	80.0	2/20	10.0	< 0.001

In OAC, CpG island promoter methylation was observed, in descending order, in 85.9% (73/85) for *NDRG4*, 80.0% (65/85) for *TFPI2*, 75.3% (64/85) for *RUNX3*, 72.9% (62/85) for *MGMT*, 55.4% (46/83) for *CHFR*, 31.8% (21/66) for *CDKN2A,* 21.2% (18/85) for *MLH1* and 12.1% (7/58) for *RASSF1* (Table 2). In OSCC, CpG island promoter methylation frequencies were lower than in OAC, which reached statistical significance for *CHFR* (25% vs 55.4%, *P* = 0.015), *TFPI2* (10% vs 80%, *P* < 0.001), *RUNX3* (40% vs 75.3%, *P* = 0.002) and *NDRG4* (5% vs 85.9%, *P* < 0.001). *CDKN2A* methylation was mutually exclusive with *CDKN2A* deletion. There were no significant associations between promoter methylation of these selected genes and clinical N or T stage in both OAC and OSCC. *CHFR* methylation was associated with an older age at diagnosis in OSCC (median 70 vs 65 years, *P* = 0.019).

### 3.4 Genomic alterations and histopathological response

**Fig. 2:**
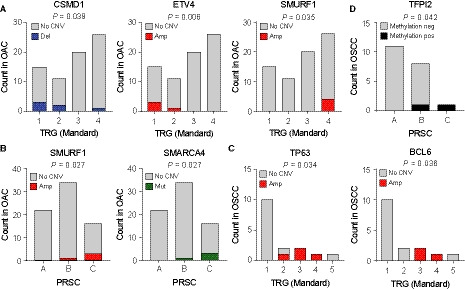
(Epi)genetic alterations in relation to histopathological response to neoadjuvant chemoradiotherapy in patients with oesophageal cancer. (A, B) Associations between (epi)genetic alterations and histopathological response in oesophageal adenocarcinoma (OAC). (A) *CSMD1* deletion and *ETV4* amplification were associated with a favourable tumour regression grade (TRG), whereas *SMURF1* amplification was associated with an unfavourable TRG in OAC. (B) *SMURF1* amplification and *SMARCA4* mutation were associated with an unfavourable prognostic score (PRSC) in OAC. (C, D) Associations between (epi)genetic alterations and histopathological response in oesophageal squamous cell carcinoma (OSCC). (C) *TP63* and *BCL6* amplification (both on chromosomal region 3q27.3‐28) were associated with an unfavourable TRG in OSCC. (D) *TFPI2* promoter methylation was associated with an unfavourable PRSC in OSCC. Linear‐by‐linear, exact test.

Since OAC and OSCC are molecularly distinct and respond differently to CRT, we analysed associations between molecular alterations and therapy response for both histological subtypes separately. The undifferentiated carcinoma was excluded from this analysis. We first evaluated recurring CNVs (≥ 5% of all samples) in relation to histopathological response according to the Mandard TRG. Thereby, we identified that within OAC deletion of *CUB and Sushi multiple domains 1, CSMD1* (8p23.2; 8.0%, 6/75) and amplification of *ETS Variant Transcription Factor 4*, *ETV4* (17q21.31; 5.3%, 4/75) were associated with a favourable Mandard TRG (*P* = 0.039 and *P* = 0.006, respectively; Fig. 2A and Table S3). Five out of six patients with *CSMD1* deletion had a Mandard TRG of 1 or 2; and all four patients with *ETV4* amplification had a Mandard TRG of 1 or 2. Amplification of *SMAD Specific E3 Ubiquitin Protein Ligase 1, SMURF1* (7q22.1; 5.3%, 4/75) on the other hand, was associated with an unfavourable Mandard TRG (*P* = 0.035); all patients with *SMURF1* amplification had a TRG 4. Due to the low frequency of *ETV4* amplifications and *CSMD1* deletions (and their co‐occurrence in one patient), they could not be confirmed as independent predictors of Mandard TRG by multiple regression analysis. In addition to the association with an unfavourable Mandard TRG, amplification of *SMURF1* was also associated with an unfavourable PRSC (*P* = 0.027; Fig. 2B).

With regard to gene mutations, only *SMARCA4* mutation (5.3%, 4/75; all missense) was associated with an unfavourable PRSC in OAC (*P* = 0.027; Fig. 2B, Table S3) but not an unfavourable Mandard's TRG, which can be explained by the difference in ypN positivity (80% vs. 38%) between OAC patients with mutant *SMARCA4* compared to wild‐type *SMARCA4*, which is not included in the Mandard's TRG.

In OSCC, amplification of chromosomal region 3q27.3‐28, harbouring *TP63* (25.0%, 4/16) and *BCL6* (18.8%, 3/16), was associated with an unfavourable Mandard TRG (*P* = 0.034 and *P* = 0.036, respectively; Fig. 2C and Table S4). There were no significant associations between gene mutations and histopathological response in OSCC.

For CpG island promoter methylation, we observed a trend towards an unfavourable Mandard TRG for *NDRG4* promoter methylation in OAC (*P* = 0.050; Table S5). In OSCC, *TFPI2* promoter methylation (10%, 2/20) was associated with an unfavourable PRSC (*P* = 0.042; Fig. 2D, Table S6), which was mostly due to all patients with *TFPI2* promoter methylation having ypN positivity (*P* = 0.032).

We did not find significant associations between histopathological response (Mandard TRG and PRSC) and disruption of specific pathways such as the RTK/RAS/PI(3)K pathway, chromatin remodelling, cell cycle, cell differentiation and proliferation; or potentially targetable genes (Fig. S5).

### 3.5 Prognostic value of molecular alterations

**Fig. 3:**
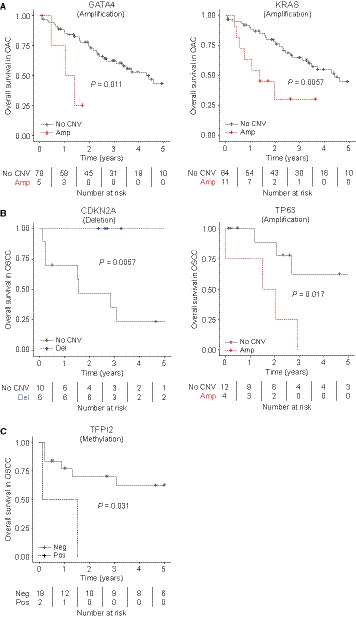
(Epi)genetic alterations in relation to survival in patients with (A) oesophageal adenocarcinoma (OAC) and (B, C) oesophageal squamous cell carcinoma (OSCC). (A) *GATA4* and *KRAS* amplification was associated with a shorter overall survival (OS) in patients with OAC. (B) In patients with OSCC, *TP63* amplification was associated with a shorter OS, whereas deletion of *CDKN2A* was associated with a longer OS. (C) Patients with OSCC and *TFPI2* promoter methylation had a shorter OS. Log‐rank test.

Next, we analysed associations between genomic and epigenetic alterations and survival. Thereby, we identified that for OAC, amplification of *KRAS* (14.7%, 11/75) and the 8p23.1 chromosomal region, harbouring *GATA4* (6.7%, 5/75), *NEIL2* (6.7%, 5/75) and *CTSB* (5.3%, 4/75), were associated with a shorter OS (median nonamplified vs amplified, 4.4 vs 1.4 years, *P* = 0.0057, HR 3.2 for *KRAS*; 4.3 vs 1.1 years, *P* = 0.011, HR 4.4, for *GATA4*; Fig. 3A, Table S3 and Fig. S6), but not DFS. Despite their distant chromosomal location, *GATA4* amplification coincided in four out of five cases with *KRAS* amplification; hence, they could not be identified as independent prognostic factors.

Additionally, associations between (epi)genetic events and an exceptionally early recurrence, that is recurrence within 1 year, were tested. In OAC, *CCND1* amplification (8%, 6/75) was associated with recurrence within one year (*P* = 0.045). There were no significant associations between CpG island promoter methylation of the selected genes and survival in OAC.

In OSCC, amplification of *TP63* (25.0%, 4/16) was associated with a shorter DFS (median nonamplified vs amplified, not reached vs 1.5 years, *P* = 0.017, HR 2.6; Fig. 3B and Table S4), which is in line with the significant association between *TP63* amplification and an unfavourable Mandard TRG. Furthermore, deletion of *CDKN2A* (37.5%, 6/16) was associated with a longer OS [median nondeleted vs deleted, 1.6 years vs not reached, *P* = 0.0057, *q* (*P*‐value corrected for multiple comparisons) = 0.0419, HR 0.015].

For CpG island promoter methylation in OSCC, *TFPI2* promoter methylation was associated with worse OS (median unmethylated vs methylated, 5.8 vs 0.1 years, *P* = 0.031; Fig. 3C). Since *CDKN2A* deletion was associated with a long OS, we tested the effect of *CDKN2A* deletion or promoter methylation on survival and found no significant associations. There were no significant associations between genomic and epigenetic alterations and recurrence within 1 year within the OSCC cohort.

Except for the association between *CDKN2A* deletion and a favourable OS in OSCC, none of the described associations with histopathological response or survival maintained significance after correction for multiple comparisons.

## 4 Discussion

Oesophageal cancer (OC) is a deadly disease, and incidence rates, especially of adenocarcinoma, are on the rise [1]. Despite a survival increment due to the addition of neoadjuvant chemoradiotherapy (CRT) to surgical resection for stage II/III disease [3], success of neoadjuvant treatment varies greatly between patients. In order to improve our understanding of treatment response and in search for biomarkers for patient selection, we performed molecular analyses on pretreatment biopsies and identified several interesting associations.

We first showed feasibility of this approach by identifying previously described genomic and epigenetic alterations in comparable frequencies in both OAC and OSCC using (mostly formalin‐fixed paraffin‐embedded) biopsies. In OAC however, none of the highly recurrent alterations such as *TP53* mutation, *ERBB2* amplification, *CDKN2A* deletion or mutation, *KRAS* amplification, and *GATA6* amplification, were associated with histopathological response to neoadjuvant CRT. Instead, we found associations involving relatively rare genetic alterations: deletion of complement inhibitor *CSMD1* (8p23.2) and amplification of transcription factor *ETV4* (17q21.31) were associated with a favourable Mandard TRG, and amplification of E3 ubiquitin ligase *SMURF1* (7q22.1) was associated with an unfavourable Mandard TRG. *SMURF1* amplification was also associated with an unfavourable PRSC, as was mutation of SWI/SNF component *SMARCA4* (BRG1).

Beyond the need to validate these associations in additional larger cohorts to determine its reproducibility, it is not clear whether these genes are really associated with CRT resistance or sensitivity or whether these genes are mere innocent bystanders. *SMARCA4* mutation and *SMURF1* amplification have been associated with a poor prognosis in gastro‐oesophageal adenocarcinoma before, potentially confirming a more aggressive phenotype, but the same accounts for *ETV4* amplification and *CSMD1* deletion [18, 19, 20, 21, 22]. Furthermore, inactivation of SMARCA4, the catalytic subunit of the SWI/SNF chromatin remodelling complex, has been linked to impaired nucleotide excision repair (NER) [23] and loss of Rb activity [24], and thereby increased platinum sensitivity in HNSCC and NSCLC cell lines [23] and NSCLC patients [25], which contrasts our findings of resistance to platinum‐containing CRT.

For ubiquitin ligase SMURF1, no association with resistance to CRT has been described before. However, as SMURF1 induces degradation of several pro‐apoptotic proteins [26], one could hypothesize that amplification of *SMURF1* disturbs the effect of CRT by preventing adequate execution of apoptosis [27]. Also for amplification of *ETV4* and deletion of *CSMD1*, no association with response to therapy has been described before, but as inducer of cyclin D3 [28] and cyclin D1 [29] upregulation, and p21 downregulation [30], *ETV4* amplification might contribute to CRT sensitivity by promoting cell cycle progression through potentially radiosensitive phases of the cell cycle. Lastly, CSMD1 is a membrane‐bound complement inhibitor [31, 32], whose tumour‐suppressing properties have been linked to its short cytoplasmic tail that contains a tyrosine phosphorylation site [32]. In gastric cancer cells, *CSMD1* downregulation has been associated with increased NF‐κB signalling, upregulation of c‐Myc and CCND1, and downregulation of E‐cadherin [22]. CSMD1 has been shown to inhibit the deposition of complement factors C3b and C9 on ovarian cancer cells and promote the degradation of C3b [32], thereby potentially inhibiting an antitumour immune response. Conversely, knockdown of *CSMD1* expression has been shown to increase the deposition of C3b on breast cancer cells [32]. The increased complement deposition on tumour cells due to CSMD1 deletion might be the link to a favourable Mandard TRG, but this needs further investigation.

In terms of survival, we did find some intriguing associations. Amplification of *KRAS* and *GATA4* was significantly associated with a shorter overall survival (OS) in our OAC cohort. Amplification of *GATA4* has already been identified as a poor prognosticator in OAC in at least two independent studies [9, 33]. Also, amplification of *KRAS* was previously found to be significantly associated with lymph node metastasis and poor OS in OAC patients treated with upfront resection [34]. Taken together, these data indicate *GATA4* and *KRAS* as promising biomarkers for early disease recurrence, which needs further investigation in prospective biomarkers studies.

In OSCC, we identified several associations between recurrent genomic alterations and response to CRT. Amplification of *TP63* was associated with an unfavourable Mandard TRG and a shorter disease‐free survival (DFS). *TP63*, which encodes p53‐related p63, is a transcription factor which overexpression has been associated with resistance to radiotherapy in oral and cervical SCC [35, 36], and conversely, p63 knockout has been shown to prevent apoptosis in noncancerous cells [37]. Interestingly, deletion of *CDKN2A* (p16^INK4a^) was strongly associated with a favourable OS in our cohort, which contrasts other reports about *CDKN2A* loss and a poor prognosis [38], including other squamous cell carcinomas [39, 40]. Although this controversy can potentially be explained by the effect of CRT in our study, this finding needs further investigation. Additionally, *TFPI2* promoter methylation was significantly associated with both an unfavourable PRSC and poor OS. *TFPI2* inhibits extracellular matrix (ECM) proteinases such as matrix metalloproteinases (MMPs), and thereby angiogenesis and invasive ability in OSCC cell lines, but its role in response to CRT has not been investigated before.

To our knowledge, this is the first publication on (epi)genetic profiling of pretreatment biopsies in relation to response to neoadjuvant CRT and survival in oesophageal cancer. With 75 and 85 OAC patients for genomic and methylation analyses, our OAC cohort was of reasonable size, and some potentially interesting associations with response to CRT were identified. The prevalence of these response‐associated alterations, however, was low, which limits their suitability as biomarker for patient selection. None of the more prevalent genetic alterations such as amplification of *ERBB2*, *EGFR*, *KRAS* or *GATA4* were enriched in one of the response groups. Therefore, we are not convinced that targeted next‐generation sequencing of pretreatment biopsies in OC will be practice‐changing. Although other factors such as immune cells or stromal components might have a bigger impact on success of CRT [41, 42] than the tumour genome, our slightly disappointing results might be the result of intratumoral genomic heterogeneity; a hallmark of OACs [12, 43, 44]. Using multiregion sequencing of primary OACs, we have previously identified significant differences within the primary tumour, including discrepancies in potentially clinically relevant alterations [12]. This intratumoral heterogeneity not only complicates representative tumour sampling, it also induces an heterogeneous treatment response [43, 45, 46, 47, 48]. Therefore, approaches such as assessment of circulating cell‐free DNA (cfDNA), which is shed by all tumour cells, may provide a more comprehensive view of the genomic landscape of OACs. However, sensitivity for cfDNA is still limited, especially in a setting without distant metastatic spread [12, 49]. Improvements in cfDNA sequencing technology could provide opportunities to detect alterations more accurately and on a larger scale than in the current study, while circumventing possible sampling bias caused by tumour heterogeneity.

## 5 Conclusions

In conclusion, this study found low‐prevalent candidate (epi)genetic biomarkers associated with response to neoadjuvant chemoradiotherapy in patients with localized oesophageal cancer. These findings may assist approaches to further individualize treatment.

## Conflict of interest

The authors declare no conflict of interest.

## Author contributions

LKK and RSAG isolated DNA, made the clinical database, performed the statistical analyses and wrote the manuscript; NCTG and AFS evaluated histopathological specimen; JWS, AC, JS, RTK, MAJMJ, RLJW and DLP provided tissue and clinical data; ME performed methylation‐specific PCR analyses; ART performed targeted sequencing; HMWV and VLJLT study oversight and feedback; AJB and SD designed and coordinated this study and wrote the manuscript.

### Peer Review

The peer review history for this article is available at https://publons.com/publon/10.1002/1878‐0261.12907.

## Supporting information


**Fig. S1.** Overall survival and disease‐free survival by histology in oesophageal cancer patients treated with neoadjuvant chemoradiotherapy followed by surgery.
**Fig. S2.** Associations between histopathological response grading systems and survival.
**Fig. S3.** Venn diagram of samples used for the custom upper gastrointestinal cancer‐specific targeted sequencing (‘OncoPanel’) vs. the promoter methylation analyses.
**Fig. S4.** Methylation index by histological subtype.
**Fig. S5.** Targetable events in relation to histopathological response to neoadjuvant chemoradiotherapy.
**Fig. S6.** Additional Kaplan Meier curves: *NEIL2* and *CTSB* (colocalized with *GATA4* on 8p23.1) amplification are associated with shorter overall survival in patients with oesophageal adenocarcinoma (OAC).

**Table S1.** Custom upper gastrointestinal cancer‐specific targeted next‐generation DNA sequencing panel overview.
**Table S2.** Baseline characteristics comparing patients whose biopsies were used with those whose biopsies were not used for custom GI‐specific next‐generation targeted sequencing.
**Table S3.** Genomic alterations and associations with response to neoadjuvant chemoradiotherapy in patients with oesophageal adenocarcinoma.
**Table S4.** Genomic alterations and associations with response to neoadjuvant chemoradiotherapy in patients with oesophageal squamous cell carcinoma.
**Table S5.** Gene promoter methylation status and association with response to neoadjuvant chemoradiotherapy in patients with oesophageal adenocarcinoma.
**Table S6.** Gene promoter methylation status and association with response to neoadjuvant chemoradiotherapy in patients with oesophageal squamous cell carcinoma.

## Data Availability

Data supporting the findings of this study are available within the article and its supplementary files. Targeted sequencing data are available via the corresponding author.
