# Supplementary material for: Molecular profiles of response to neoadjuvant chemoradiotherapy in oesophageal cancers to develop personalized treatment strategies
Source: Mol Oncol. 2021 Feb 23;15(4):901–14. doi: 10.1002/1878-0261.12907 (PMC8024738; doi:10.1002/1878-0261.12907)
Supplement: Supplementary file 1 — Fig. S1. Overall survival and disease‐free survival by histology in oesophageal cancer patients treated with neoadjuvant chemoradiotherapy followed by surgery. Fig. S2. Associations between histopathological response grading systems and survival. Fig. S3. Venn diagram of samples used for the custom upper gastrointestinal cancer‐specific targeted sequencing (‘OncoPanel’) vs. the promoter methylation analyses. Fig. S4. Methylation index by histological subtype. Fig. S5. Targetable events in relation to histopathological response to neoadjuvant chemoradiotherapy. Fig. S6. Additional Kaplan Meier curves: NEIL2 and CTSB (colocalized with GATA4 on 8p23.1) amplification are associated with shorter overall survival in patients with oesophageal adenocarcinoma (OAC). [file MOL2-15-901-s001.pdf]

Figure S1

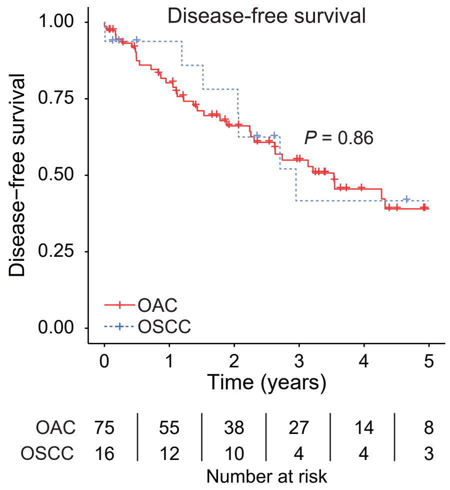

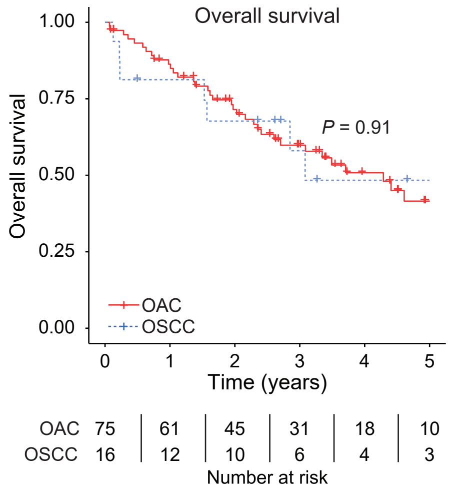

Population (*N*)	Median DFS (years) (95% CI)	1-year DFS rate (%) (95% CI)	5-year DFS rate (%) (95% CI)
Overall (91)	3.21 (2.62-NA)	82.4 (74.6-90.9)	40.0 (29.2-54.8)
OAC (75)	3.53 (2.31-NA)	80.2 (71.5-90.1)	39.0 (27.1-56.1)
OSCC (16)	2.95 (2.05-NA)	93.8 (82.6-100)	41.7 (20.6-84.3)

Population (*N*)	Median OS (years) (95% CI)	1-year OS rate (%) (95% CI)	5-year OS rate (%) (95% CI)
Overall (91)	4.29 (2.85-NA)	85.3 (78.3-93.0)	43.1 (32.1-58.0)
OAC (75)	4.29 (2.70-NA)	86.3 (78.8-94.6)	41.5 (29.4-58.6)
OSCC (16)	3.08 (1.57-NA)	81.2 (64.2-100)	48.4 (27.0-86.6)

Figure S2A,B

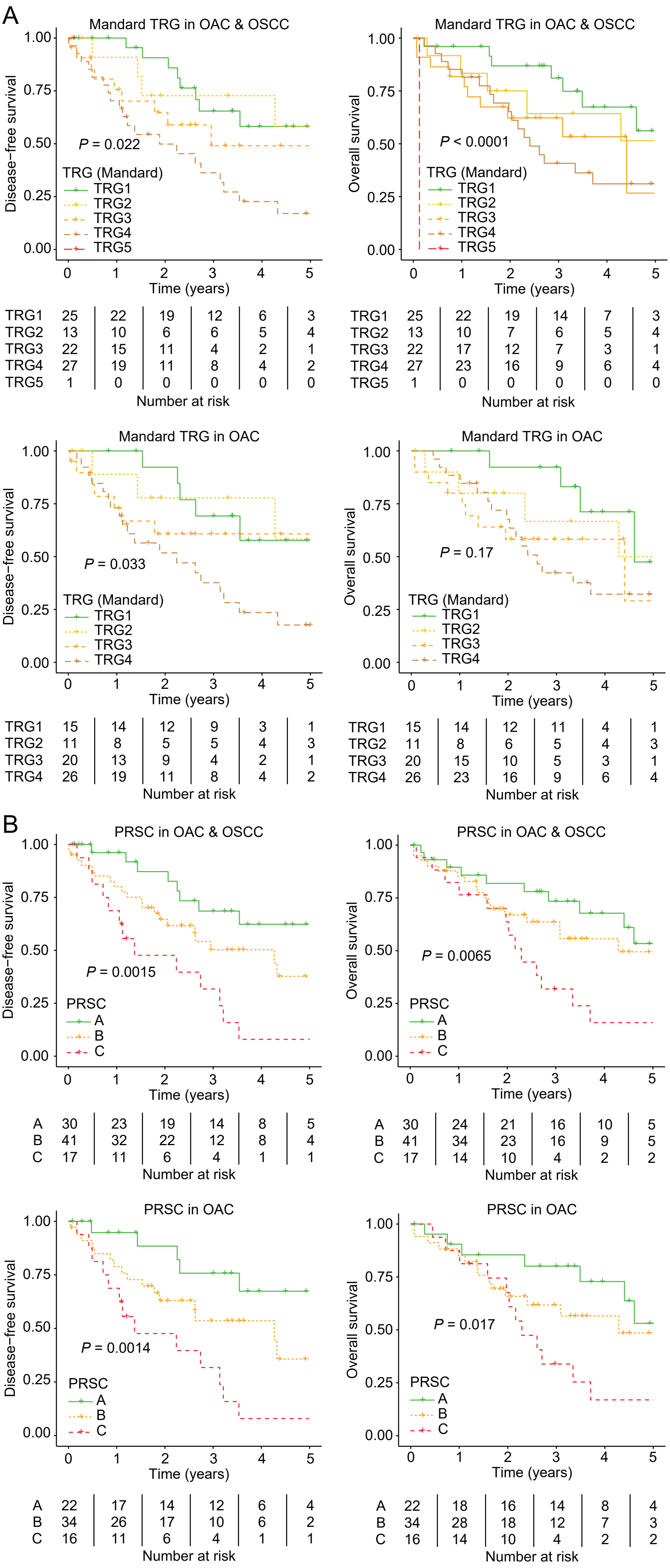
A
Mandard TRG in OAC & OSCC
Disease−free survival
P = 0.022
TRG (Mandard)
TRG1
TRG2
TRG3
TRG4
TRG5
Time (years)
TRG1 25 22 19 12 6 3
TRG2 13 10 6 6 5 4
TRG3 22 15 11 4 2 1
TRG4 27 19 11 8 4 2
TRG5 1 0 0 0 0 0
Number at risk
Mandard TRG in OAC & OSCC
Overall survival
P < 0.0001
TRG (Mandard)
TRG1
TRG2
TRG3
TRG4
TRG5
Time (years)
TRG1 25 22 19 14 7 3
TRG2 13 10 7 6 5 4
TRG3 22 17 12 7 3 1
TRG4 27 23 16 9 6 4
TRG5 1 0 0 0 0 0
Number at risk
Mandard TRG in OAC
Disease−free survival
P = 0.033
TRG (Mandard)
TRG1
TRG2
TRG3
TRG4
Time (years)
TRG1 15 14 12 9 3 1
TRG2 11 8 5 5 4 3
TRG3 20 13 9 4 2 1
TRG4 26 19 11 8 4 2
Number at risk
Mandard TRG in OAC
Overall survival
P = 0.17
TRG (Mandard)
TRG1
TRG2
TRG3
TRG4
Time (years)
TRG1 15 14 12 11 4 1
TRG2 11 8 6 5 4 3
TRG3 20 15 10 5 3 1
TRG4 26 23 16 9 6 4
Number at risk
B
PRSC in OAC & OSCC
Disease−free survival
P = 0.0015
PRSC
A
B
C
Time (years)
A 30 23 19 14 8 5
B 41 32 22 12 8 4
C 17 11 6 4 1 1
Number at risk
PRSC in OAC & OSCC
Overall survival
P = 0.0065
PRSC
A
B
C
Time (years)
A 30 24 21 16 10 5
B 41 34 23 16 9 5
C 17 14 10 4 2 2
Number at risk
PRSC in OAC
Disease−free survival
P = 0.0014
PRSC
A
B
C
Time (years)
A 22 17 14 12 6 4
B 34 26 17 10 6 2
C 16 11 6 4 1 1
Number at risk
PRSC in OAC
Overall survival
P = 0.017
PRSC
A
B
C
Time (years)
A 22 18 16 14 8 4
B 34 28 18 12 7 3
C 16 14 10 4 2 2
Number at risk

Figure S2C

C

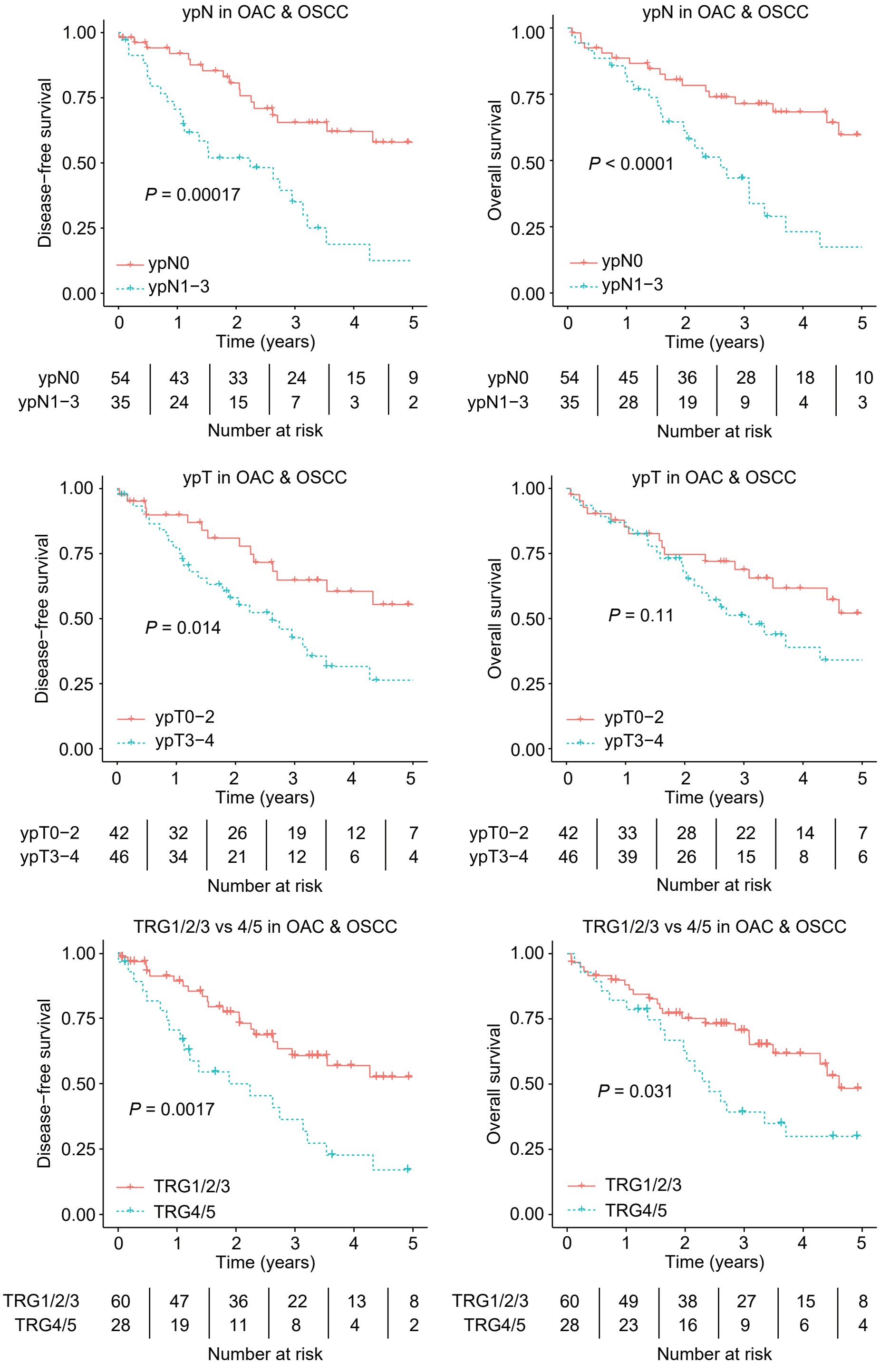

ypN in OAC & OSCC
Disease−free survival
P = 0.00017
ypN0
ypN1−3
Time (years)
ypN0 54 43 33 24 15 9
ypN1−3 35 24 15 7 3 2
Number at risk
ypN in OAC & OSCC
Overall survival
P < 0.0001
ypN0
ypN1−3
Time (years)
ypN0 54 45 36 28 18 10
ypN1−3 35 28 19 9 4 3
Number at risk
ypT in OAC & OSCC
Disease−free survival
P = 0.014
ypT0−2
ypT3−4
Time (years)
ypT0−2 42 32 26 19 12 7
ypT3−4 46 34 21 12 6 4
Number at risk
ypT in OAC & OSCC
Overall survival
P = 0.11
ypT0−2
ypT3−4
Time (years)
ypT0−2 42 33 28 22 14 7
ypT3−4 46 39 26 15 8 6
Number at risk
TRG1/2/3 vs 4/5 in OAC & OSCC
Disease−free survival
P = 0.0017
TRG1/2/3
TRG4/5
Time (years)
TRG1/2/3 60 47 36 22 13 8
TRG4/5 28 19 11 8 4 2
Number at risk
TRG1/2/3 vs 4/5 in OAC & OSCC
Overall survival
P = 0.031
TRG1/2/3
TRG4/5
Time (years)
TRG1/2/3 60 49 38 27 15 8
TRG4/5 28 23 16 9 6 4
Number at risk

Figure S3

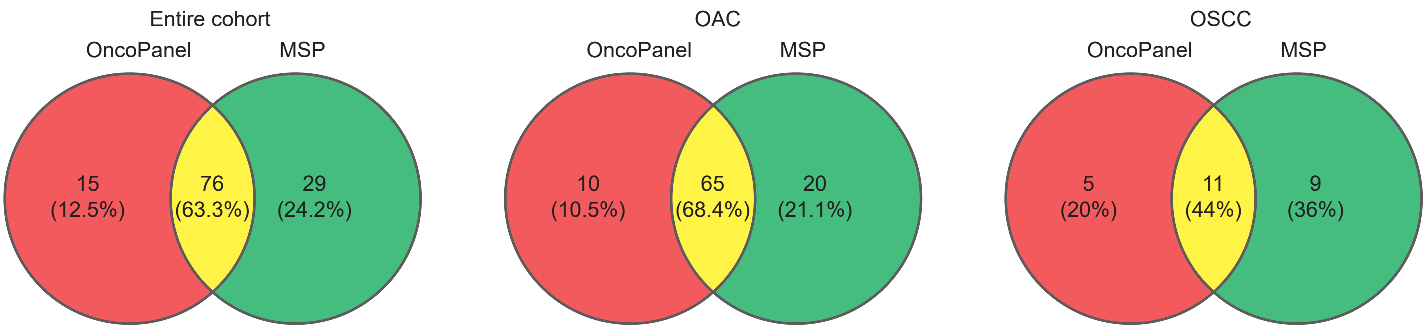
Entire cohort
OncoPanel
MSP
15
(12.5%)
76
(63.3%)
29
(24.2%)
OAC
OncoPanel
MSP
10
(10.5%)
65
(68.4%)
20
(21.1%)
OSCC
OncoPanel
MSP
5
(20%)
11
(44%)
9
(36%)

Figure S4

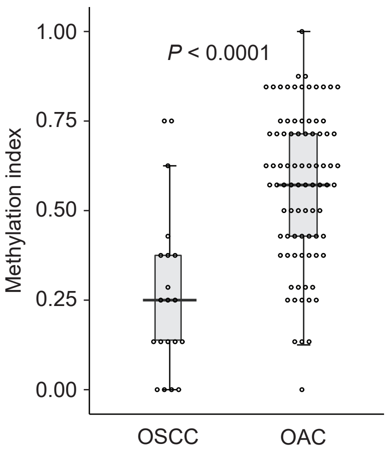

Figure S5

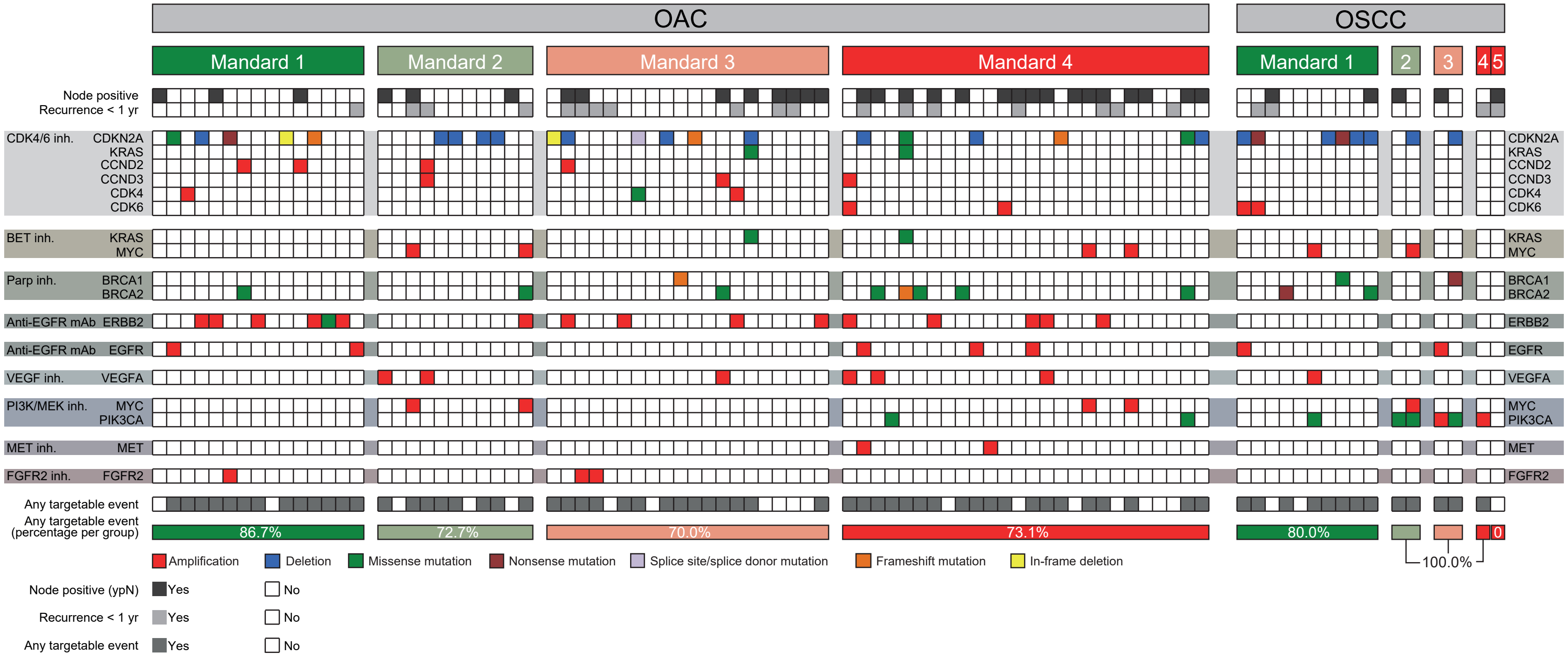
OAC
OSCC
Mandard 1
Mandard 2
Mandard 3
Mandard 4
Mandard 1
2
3
4
5
Node positive
Recurrence < 1 yr
CDK4/6 inh.
CDKN2A
KRAS
CCND2
CCND3
CDK4
CDK6
BET inh.
KRAS
MYC
Parp inh.
BRCA1
BRCA2
Anti-EGFR mAb
ERBB2
Anti-EGFR mAb
EGFR
VEGF inh.
VEGFA
PI3K/MEK inh.
MYC
PIK3CA
MET inh.
MET
FGFR2 inh.
FGFR2
Any targetable event
Any targetable event (percentage per group)
86.7%
72.7%
70.0%
73.1%
80.0%
0
100.0%
Amplification
Deletion
Missense mutation
Nonsense mutation
Splice site/splice donor mutation
Frameshift mutation
In-frame deletion
Node positive (ypN)
Yes
No
Recurrence < 1 yr
Yes
No
Any targetable event
Yes
No

Figure S6

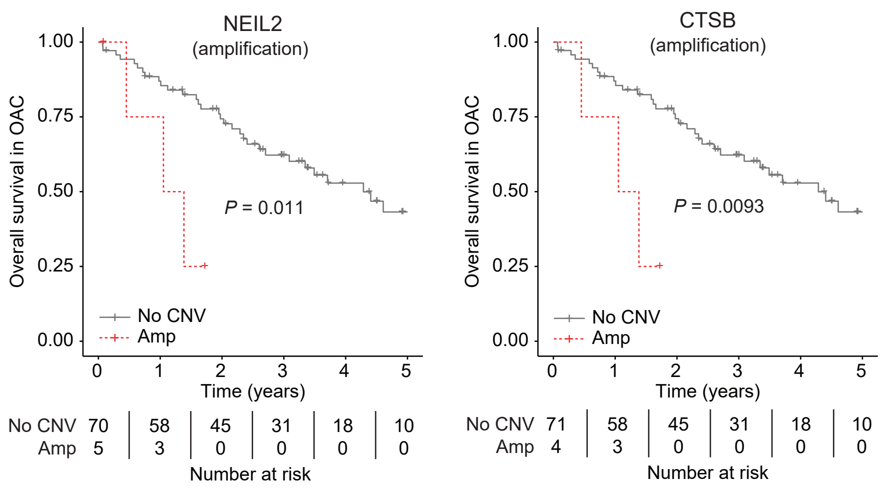
NEIL2
(amplification)
Overall survival in OAC
1.00
0.75
0.50
0.25
0.00
P = 0.011
No CNV
Amp
0
1
2
3
4
5
Time (years)
No CNV 70 58 45 31 18 10
Amp 5 3 0 0 0 0
Number at risk
CTSB
(amplification)
Overall survival in OAC
1.00
0.75
0.50
0.25
0.00
P = 0.0093
No CNV
Amp
0
1
2
3
4
5
Time (years)
No CNV 71 58 45 31 18 10
Amp 4 3 0 0 0 0
Number at risk
